# Dose of the Extract of Conium

**Published:** 1845-06-01

**Authors:** 


Dose of the Extract of Conium.—Dr. Woodward, Physician to the State, Lunatic Hospital at Worcester, Mass. in his annual report for 1844, says of the Conium maculatum, " It is only in large doses that it is useful in any case. The minimum dose is ten grains three times a day ; the maximum dose two, three, or four drachms, frequently repeated. I have rarely found any advantage from doses less than fifteen or twenty grains, three or four times a day, but, ordinarily, give from thirty to forty grains, as frequently repeated." See Am. Journal Med. Sciences, April, 1845.

These doses are so much greater than those directed in our standard works on Materia Medica, and so different, in this respect, from the usual prescriptions of the profession, that we fear there is some mistake. We apprehend that Dr. W. has been unfortunate in the choice of his extracts. Extracts are frequently dispensed by apothecaries, which are prepared by the *Shakers* by simply evaporating water in which the plant has been boiled. They are very different from the inspissated juice. In our own experience, we have derived no satisfactory results from the use of this drug, excepting when we have obtained the English extract, and it is necessary that this be very carefully selected. We should hesitate before venturing to prescribe the English article in the doses recommended by Dr. W. until assured what his experience has been with it.

